# Achilles Allograft Reconstruction for Chronic Pectoralis Major Rupture: A Case Report

**DOI:** 10.7759/cureus.91253

**Published:** 2025-08-29

**Authors:** Benjamin Blitz, Connor J O'Leary, Pasquale Gencarelli, James M Lee Jr., Rahul Mittal

**Affiliations:** 1 Department of Orthopedic Surgery, Rutgers Robert Wood Johnson Medical School, New Brunswick, USA; 2 Department of Orthopedic Surgery, New York University (NYU) Langone Health, New York City, USA; 3 Department of Orthopedic Surgery, Orange Orthopaedic Associates, West Orange, USA; 4 Depatment of Health Informatics, Rutgers University, Piscataway, USA

**Keywords:** achilles allograft, achilles allograft reconstruction, chronic pectoralis rupture, pectoralis major reconstruction, pectoralis major rupture

## Abstract

Managing a chronic rupture of the pectoralis major muscle (PMM) presents notable challenges. Delayed presentation following the initial injury often necessitates surgical intervention to restore both functional capacity and aesthetic appearance. In chronic cases, where tendon retraction and compromised tissue quality are common, surgical repair with graft augmentation is typically required. We report the case of a 41-year-old male who presented four months after sustaining a left pectoralis major rupture while lifting at work. Due to the chronic nature of the injury and retraction of the tendon, primary repair was not feasible. Surgical reconstruction was performed using an Achilles tendon allograft. At eight months postoperatively, the patient demonstrated satisfactory progress, with only mild deficits in range of motion, strength, and cosmetic appearance. He continued to engage in a structured rehabilitation program, beginning with immobilization and passive range-of-motion exercises, followed by progressive strengthening over several months. This case highlights the successful use of Achilles tendon allograft in the surgical reconstruction of a chronic pectoralis major rupture and contributes to the current body of literature supporting this approach.

## Introduction

Although uncommon, ruptures of the pectoralis major muscle (PMM) are increasingly recognized, particularly among high-intensity athletes and manual laborers. The pectoralis major is a large, fan-shaped muscle of the anterior chest wall that plays a key role in shoulder adduction, internal rotation, and flexion. It consists of two heads, the clavicular and sternocostal heads, which converge into a single tendon that inserts on the lateral lip of the bicipital groove of the humerus. Its anatomical structure and functional significance make it essential for many activities involving pushing or lifting, and injury to this muscle can result in significant functional impairment.

Multiple reviews indicate that the majority of reported PMM rupture cases have emerged within the past 20-30 years, with a notable rise since the 1990s [1,2]. Imaging studies and surgical case series from the last decade continue to report an increasing incidence, especially in young, active males engaged in physically demanding activities, including professional sports and military service [3-5]. This trend is largely attributed to heightened awareness and improved diagnostic capabilities, as well as broader participation in high-intensity resistance training and competitive athletics [6].

Chronic PMM ruptures are defined as those diagnosed more than eight weeks after injury and are associated with significant surgical challenges, including tendon retraction, fibrosis, and muscle degeneration [7]. These injuries are often missed during the acute phase, leading to delayed diagnosis and deterioration of tissue quality, which can make direct tendon repair technically difficult or impossible [8]. Surgical management strategies vary, ranging from primary suture repair in acute cases to more complex reconstructions in chronic cases, utilizing autografts or allografts. Autografts such as semitendinosus or fascia lata have been employed in chronic reconstructions, though reported outcomes vary in terms of restoring strength, function, and cosmetic appearance [9-12].

This case report describes the surgical reconstruction of a chronic PMM rupture using an Achilles tendon allograft. While the use of Achilles allografts has gained popularity in recent years, published reports remain limited, and available outcome data are variable and often lack long-term follow-up. This case adds to the evolving body of literature on allograft-based repair techniques for chronic PMM injuries.

## Case presentation

**Figure 1:**
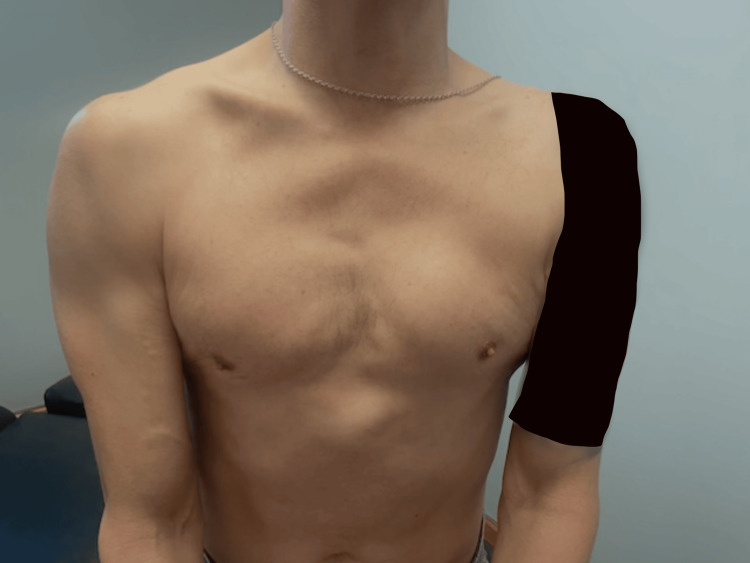
Pre-operative image demonstrating left pectoralis major muscle atrophy.

In December 2022, a 41-year-old right-hand-dominant man sustained an injury to his left arm after falling through scaffolding at work. He recalled a discrete “pop” at the time of injury but did not seek immediate medical evaluation. Over the following months, he experienced progressive weakness, cramping, and a noticeable contour deformity of the left chest (Figure 1), which began to interfere with daily activities such as lifting and pushing. The patient had no notable past medical conditions, was not on any regular medications, and reported no contributory family history.

On physical examination, there was palpable volume loss of the superior-lateral aspect of the left pectoralis major. Pectoralis strength measured 4/5 on the left versus 5/5 on the right, and the patient was tender over the bicipital groove. A well-healed surgical scar from a prior, unrelated procedure was observed near the anterior-superior acromioclavicular joint. MRI demonstrated a Grade I rupture of the left pectoralis major without frank tendon retraction, consistent with a chronic rupture. After counselling on non-operative and operative options, the patient, otherwise in excellent health, elected surgical reconstruction due to persistent cramping and functional weakness.

Operative technique

With the patient in a “lazy” beach-chair position and the arm supported by a SPIDER Limb Positioner (Smith & Nephew, Andover, MA, USA), a 12-cm deltopectoral incision was made beginning at the coracoid process and extending distally. The deltopectoral interval was developed with care to protect the cephalic vein. Dissection medially and inferiorly revealed a retracted muscle belly located approximately 10 cm medial to its humeral insertion. Because the residual tendon stump was insufficient for direct repair, an Achilles tendon allograft was selected for reconstruction.

Lateral dissection exposed the native humeral footprint, which remained largely intact. The long head of the biceps tendon was identified and preserved, and the humeral cortex was lightly skeletonized to prepare a fresh bone bed. Throughout the procedure, the cephalic vein, adjacent vascular structures, and brachial plexus elements were protected.

**Figure 2:**
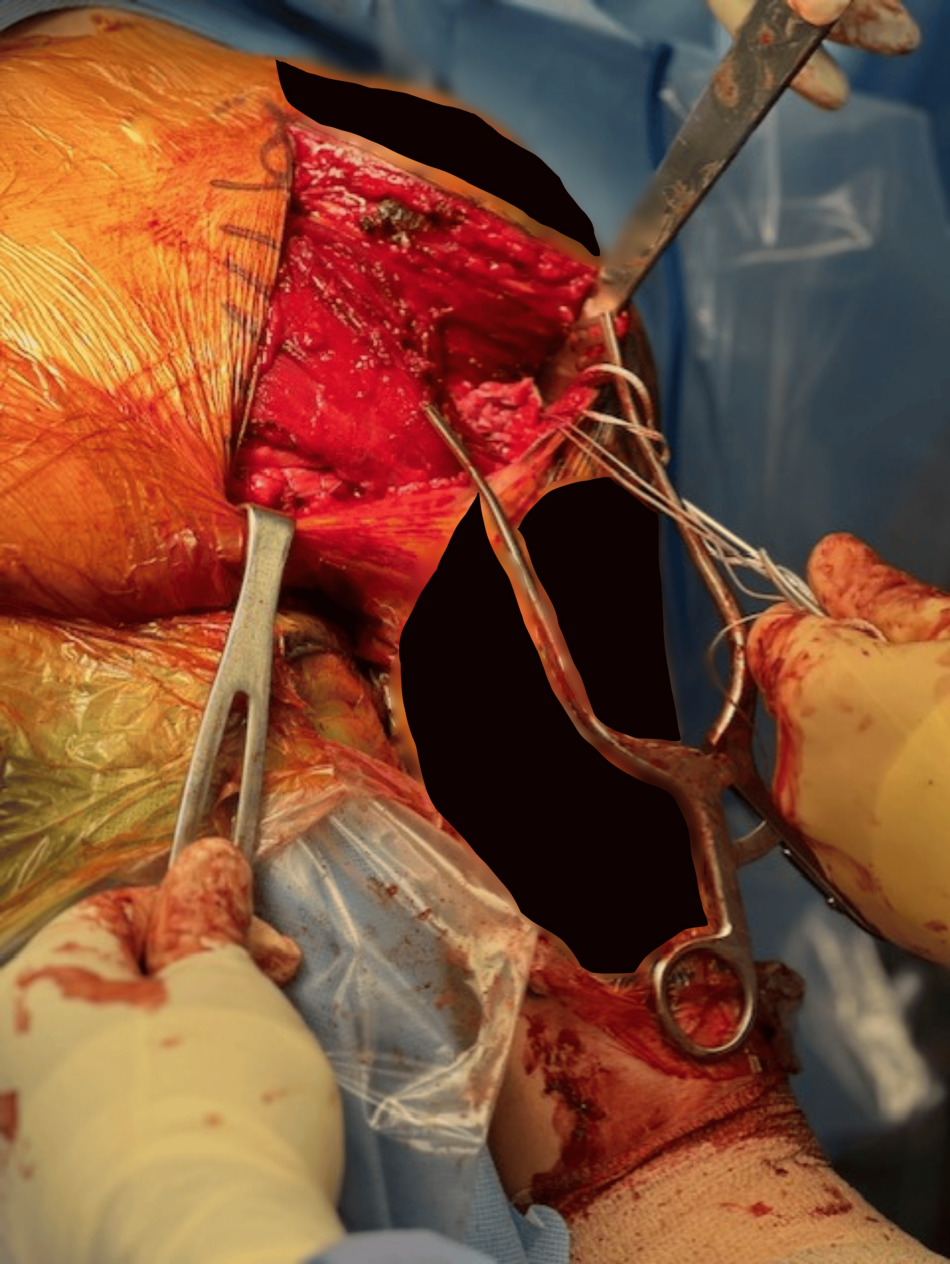
Operative image demonstrating the reconstructed pectoralis major.

Using a modified Krakow technique with #5 FiberWire (Arthrex, Naples, FL, USA) and FiberTape (Arthrex, Naples, FL, USA), the Achilles allograft was woven medially into the retracted pectoralis tendon stump. Two unicortical drill holes, spaced 1.5-2.0 cm apart, were created in the humerus. Sutures were passed through an Arthrex cortical button system, which was flipped unicortically to tension the graft and restore anatomic length (Figure 2). Supplemental anchors were applied to secure the construct; stable fixation was confirmed through passive internal and external rotation. After meticulous hemostasis, the wound was closed in layers, and the arm was placed in an immobilizing sling.

Postoperative course

The immediate postoperative period was uneventful aside from mild incisional discomfort. At five weeks, supervised physical therapy focusing on passive and active-assisted range of motion was initiated, limiting external rotation beyond 45°. By three months, the patient reported a burning sensation on overhead elevation and had discontinued formal therapy in favor of home exercises. Examination revealed a well-healed incision, active forward flexion to 140°, bilateral 5/5 strength of the biceps and pectoralis, and localized atrophy of the lateral pectoralis belly; the repair itself remained intact. Physical therapy was reinstated, and the patient was restricted to lifting no more than 15 lb.

At five months post-operation, the patient described gradual improvement with only mild-to-moderate shoulder pain. Active forward flexion, internal rotation, full hand and wrist strength, and 150° of abduction were documented. Mild residual atrophy of the left pectoralis major persisted.

Eight months after surgery, functional gains continued; however, the patient still experienced mild pain and was dissatisfied with the residual cosmetic deformity. Continued guided physical therapy was recommended to optimize strength and appearance.

## Discussion

Chronic PMM ruptures are rare and present unique surgical challenges due to tendon retraction, muscle degeneration, and the development of fibrosis, all of which complicate primary repair [13]. These injuries are typically defined as those diagnosed more than eight weeks after the initial trauma and often arise from delayed recognition or treatment. In our case, the patient presented four months post-injury, a delay that necessitated graft augmentation to bridge the retracted tendon and restore the functional anatomy of the pectoralis major.

Various graft options have been described in the literature for chronic PMM reconstruction, including autografts such as semitendinosus and fascia lata, and allografts such as acellular dermal matrix or Achilles tendon [14]. Although multiple reports describe these techniques, many employ grafts that are relatively narrow or thin, often requiring alternative configurations or tensioning strategies for adequate reconstruction [3,12,15-17]. In our patient, the significant retraction of the muscle belly and the wide gap from the humeral insertion necessitated a robust and lengthy graft. The Achilles tendon allograft was selected for its superior tensile strength and favorable handling characteristics, offering a biomechanically sound solution.

Although the use of Achilles tendon allografts in PMM reconstruction has been reported, published cases remain limited, and long-term outcomes are not well established. Our surgical technique involved a modified Krakow suture configuration with #5 FiberWire and FiberTape, along with cortical fixation using the Arthrex button system. This construct allowed for secure fixation and restoration of appropriate tension, critical considerations in chronic reconstructions where soft tissue integrity is compromised.

Postoperatively, the patient demonstrated steady functional improvement with restored shoulder strength and a near-full range of motion by eight months. While mild residual pain, muscle atrophy, and cosmetic asymmetry persisted, these did not significantly impact function. These findings are consistent with prior literature, such as the study by Merolla et al., which reported favorable functional outcomes despite aesthetic limitations following graft-based PMM reconstructions [9]. The cosmetic deformity observed in our case is likely attributable to the chronicity of the injury and the muscle atrophy that occurred during the untreated period.

Importantly, the patient experienced no major complications such as rerupture, infection, or hardware failure, supporting the safety and efficacy of Achilles allograft use in this setting. However, this case also highlights the importance of structured and continuous postoperative rehabilitation. The patient’s temporary discontinuation of physical therapy corresponded with a plateau in recovery and the onset of subjective symptoms, underscoring the critical role of supervised rehabilitation in maximizing functional outcomes after complex PMM repairs.

This case contributes to the growing body of evidence supporting the use of Achilles tendon allografts in the surgical management of chronic PMM ruptures. While further research involving larger patient cohorts and long-term follow-up is needed, our experience suggests that this technique can achieve satisfactory functional outcomes, with acceptable cosmetic trade-offs in appropriately selected patients.

## Conclusions

The management of chronic PMM ruptures remains challenging due to tendon retraction, poor tissue quality, and extensive fibrosis. In this case, surgical reconstruction using an Achilles tendon allograft was effective in restoring shoulder function, with only minor residual cosmetic deformity. Compared to narrower graft options such as hamstring autografts or dermal allografts, the Achilles tendon allograft provided superior length and tensile strength, allowing for more anatomic reconstruction and appropriate tensioning of the retracted musculotendinous unit.

Successful outcomes in chronic PMM repairs require not only technically sound surgical intervention but also structured postoperative rehabilitation. Close follow-up and adherence to guided physical therapy are critical to optimizing functional recovery and minimizing long-term deficits.

While our findings support the use of Achilles tendon allografts in this context, further research is necessary to evaluate long-term outcomes, comparative efficacy, and complication rates associated with different graft choices in chronic PMM rupture repair.
